# Highly dispersed Pd on macroporous SmMn_2_O_5_ mullite for low temperature oxidation of CO and C_3_H_8_†

**DOI:** 10.1039/c7ra11551b

**Published:** 2018-01-31

**Authors:** Yuning Zhu, Chun Du, Zijian Feng, Yongjie Chen, Hang Li, Rong Chen, Meiqing Shen, Bin Shan

**Affiliations:** State Key Laboratory of Material Processing and Die and Mould Technology, School of Materials Science and Technology, Huazhong University of Science and Technology Wuhan 430074 China bshan@mail.hust.edu.cn; State Key Laboratory of Digital Manufacturing Equipment and Technology, School of Mechanical Science and Engineering, Huazhong University of Science and Technology Wuhan 430074 China; School of Chemical Engineering and Technology, Tianjin University 92 Weijin Road, Nankai District Tianjin 300072 China

## Abstract

The catalytic behavior of a palladium catalyst supported on macroporous SmMn_2_O_5_ mullite (Pd/SMO-EG&M) for CO and C_3_H_8_ oxidation was measured under lean-burn conditions. Different analytical techniques including XRD, Raman, BET, CO chemisorption, SEM, FTEM, XPS, TPD, TPR and CO + O_2_ pulse were undertaken to evaluate its physical and chemical properties. It was concluded that the crystal structure, morphology and specific surface area (SSA) of SmMn_2_O_5_ remained unchanged after Pd addition. The Pd/SMO-EG&M exhibited a low complete transformation temperature for CO (105 °C) and C_3_H_8_ (350 °C) oxidation. Such remarkable oxidation activity was attributed to high Pd dispersion (38.4%), which improved the reducibility and mobility of oxygen species, as revealed by TPR and TPD measurements. The high activity of oxygen species for Pd/SMO-EG&M above 250 °C accelerated the oxidation capacity as well. In a word, our study indicates that the macroporous Pd–mullite catalyst has potential applications in exhaust purification for gasoline vehicle.

## Abstract 2

Pd-modified SMO mullite catalysts were synthesized and found to have excellent catalytic activity for CO and C_3_H_8_ oxidation. The remarkable oxidation activity was attributed to the high Pd dispersion.

## Graphical abstract

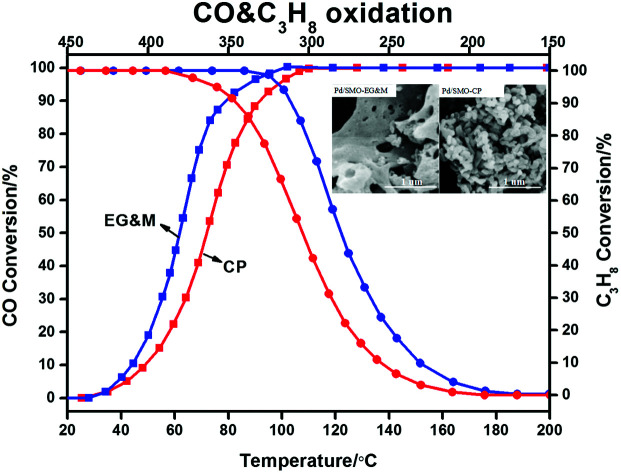

## 1. Introduction

The noxious exhaust of automobiles has become an urgent and compelling issue across the world. Specifically, because of the low oxidation efficiency of conventional three-way catalysts (TWC) below 300 °C, a large portion (50–80%) of unburned carbon monoxide (CO) and hydrocarbons (HCs) is emitted into the air during the initial cold-start transients, resulting in photochemical smog and the greenhouse effect directly.^1,2^ To meet the strict current legislation of governments for ultra low-emission or zero-emission vehicles, it is imperative to explore highly efficient catalysts at relatively low temperatures. Among various types of commercial platinum group metals (PGMs), Pd has been regarded as an active component in automotive converters to eliminate CO and other exhaust fumes.^3–5^ Owing to the interaction between the metal oxide support and the active metal species, the catalytic behavior, durability and dispersion of heterogeneous Pd catalysts are closely associated with the inherent physical and chemical properties of the support.^6–9^ So far, the simple metal oxides, such as CeO_2_, Co_3_O_4_, TiO_2_, ZrO_2_ and MnO_*x*_, have been extensively studied for their large specific surface area.^10–13^ Whereas in practical applications, metal oxide supported catalysts are unstable after redox cycling, which results in the deactivation of active Pd species.

In the last decade, perovskite-type mixed oxides have been developed as potential low-cost catalysts for diesel exhaust treatment system.^14–16^ Due to their unique thermal, mechanical and redox stability and catalytic performance, perovskites could serve as active support to reduce the noble metal loading.^17–19^ Despite their sufficient catalytic efficiency, the weak hydrothermal resistance and small oxygen storage capacity (OSC) lead to depletion of catalytic activity directly after rapid aging cycles.^20–22^ In 2012, Wang *et al.* proposed a new type of mixed-phase oxide catalyst for NO oxidation based on Mn mullite ((Sm, Gd)Mn_2_O_5_), which has exhibited favorable thermal and structural stability.^23^ Compared with the representative LaCoO_3_ perovskite catalyst, the SmMn_2_O_5_ mullite catalyst has higher TWC reactivity, anti-poisoning ability and OSC.^24^ Hence, it is probable that mullite-supported Pd catalysts should display improved performance and serve as potential candidate as low precious metal loading catalysts. Note that diffusion has been considered as the limit process for the overall performance of bulk catalysts, fabricating a porous structure is desirable to achieve high amount of surface accessible active sites and a better dispersion, ultimately stimulating its catalytic oxidation efficiency.^7,25,26^ Therefore, it is meaningful to explore Pd on hydrothermally stable porous SmMn_2_O_5_ support with high performance.

In this work, a macroporous SmMn_2_O_5_ mullite catalyst (SMO) was prepared by the combustion of organic solutions according to our previous study.^27^ Afterwards, a small fraction (0.5 wt%) of Pd was loaded on the catalyst and its catalytic activities were determined by CO and C_3_H_8_ oxidation. The physical and chemical properties of as-prepared Pd catalysts were analysed by XRD, Raman, SSA, SEM, FTEM, ICP, XPS, H_2_-TPR, O_2_-TPD and CO + O_2_ pulse studies. The catalyst exhibits superior catalytic activity and stability for CO and C_3_H_8_ oxidation due to a larger number of active sites induced by higher Pd dispersion (38.4%). The adsorbed oxygen species increases dramatically for dispersed Pd/SmMn_2_O_5_-EG&M catalyst as revealed by O_2_-TPD test. The higher catalytic property is attributed to the higher Pd dispersion, inducing stronger ability of oxygen migration and more adsorption site on the macroporous mullite as demonstrated by TPO and CO-TPD test. Meanwhile, the easier activation of available oxygen species bond confirmed by H_2_-TPR and CO + O_2_ pulse experiment accelerates the reaction as well.

## 2. Catalyst characterization

The PANalytical Xpert PRO diffractometer recorded the X-ray diffraction (XRD) spectrum from 10° to 60° in 2*θ* angle at room temperature using Cu Kα radiation (*λ* = 1.5406 Å) with a 0.02° step scan. The crystal structures were identified using the JCPDS data bank. Raman spectrum analysis was conducted on a Renishaw InVia Reflex spectrometer from 150 to 800 cm^−1^. The excitation wavelength of the laser was set at 532 nm with a spectral resolution of 1.5 cm^−1^. The surface textural parameters of each sample was measured by nitrogen adsorption using Micromeritics ASAP 2020M physisorption analyser at 77 K. Prior to the measurement, the samples were degassed in vacuum oven at 120 °C overnight. The field-emission scanning electron microscopy (FE-SEM) was performed on a Nova NanoSEM 450 microscope, and field-emission transmission electron microscopy (FE-TEM) and high-resolution transmission electron microscopy (HRTEM) images were analysed on a Tecnai G2 30 microscope. Inductively coupled plasma atomic emission spectroscopy (ICPAES) was produced by an Optical 4300DV to determine the actual mass loading of Pd. X-ray photoelectron spectroscopy (XPS) was acquired to study the chemical compositions at 10^−7^ Pa using an AXIS-ULTRA-DLD spectrometer. The binding energy (BE) of C 1s was calibrated to 284.8 eV. The adsorption properties, oxygen storage capacity (OSC) measurements and catalytic activity measurements were undertaken on an automatic adsorption instrument (FINESORB-3010E, Zhejiang Finetec Co). The detailed experimental process was given in ESI (S2†).

## 3. Results and discussion

### 3.1. Physical characterization

**Fig. 1:**
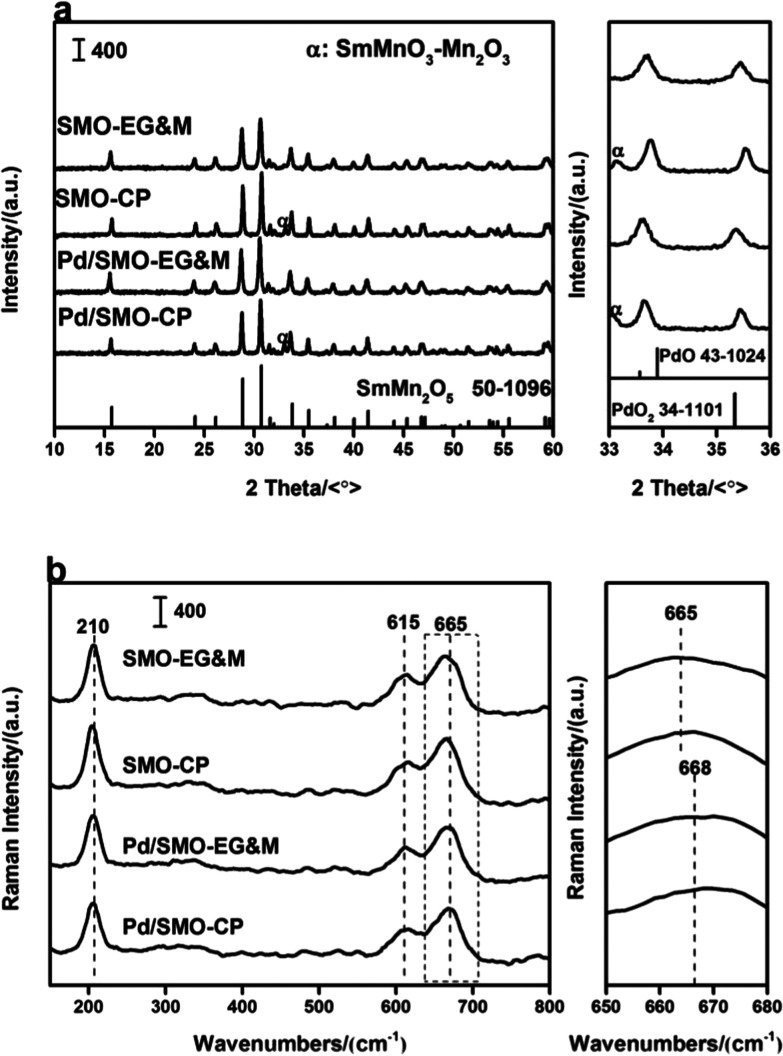
(a) XRD profile; (b) Raman spectra of the SMO and Pd/SMO.

Fig. 1a displays the XRD profiles of pristine SMO supports (SMO-EG&M and SMO-CP) and Pd loaded catalysts (Pd/SMO-EG&M and Pd/SMO-CP). All samples exhibit characteristic reflections of the single-phase orthorhombic SmMn_2_O_5_ structure (SmMn_2_O_5_ phase [52-1096]) at 29.1° and 31.2°. Specifically, additional phases of SmMnO_3_ and Mn_2_O_3_ are observed for CP samples around 32.9°, which is similar to our previous work.^28,29^ For Pd loaded catalysts, due to low content or small crystalline size, no reflections of PdO_*x*_ or metallic Pd are observed. Additionally, the Pd/SMO catalysts have similar half-height width and intensity of the typical peaks with that of SMO-EG&M and SMO-CP supports, implying negligible changes on crystallization and crystalline size after Pd addition.

The catalysts were then analysed by Raman spectroscopy to elucidate their structural features. In Fig. 1b, all samples display three characteristic bands at 210, 615 and 665 cm^−1^, respectively. On the basis of lattice dynamics calculations, the band at 210 cm^−1^ is associated with A_g_ mode of Mn–Mn translation motion along the chain direction,^26,28,30^ while the rest two pronounced bands at higher frequencies (>600 cm^−1^) correspond to the asymmetric stretching vibration of Mn–O–Mn in Mn^4+^O_6_ units.^30–33^ Moreover, the peak at 665 cm^−1^ shifts to a higher frequency after Pd addition, implying the generation of new phases. Note that the vibration mode of Pd–O bond at 672 cm^−1^ has been observed for some Pd-containing samples,^34,35^ the band at 665 cm^−1^ for the Pd/SMO can be deduced as a combination of vibration bands from mullite structure and palladium oxides.^27^ Moreover, the length and angle of Mn–O band might be influenced by the Pd addition since the lattice oxygen of mullite might be shared with Pd species, contributing to the variation of the peak at 665 cm^−1^ as well.^36,37^ Thus variation might affect the activity of lattice oxygen.

**Fig. 2:**
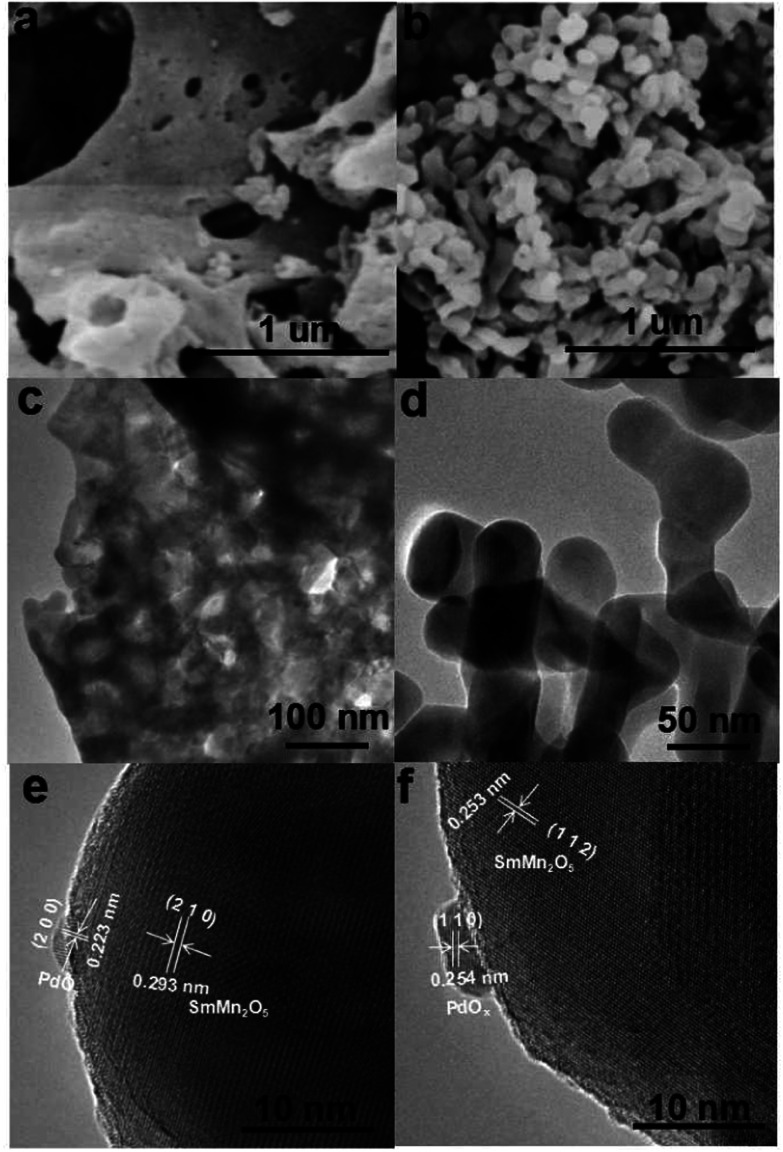
SEM, TEM and HRTEM images of (a), (c) and (e) Pd/SMO-EG&M and (b), (d) and (f) Pd/SMO-CP.

The morphologies of Pd/SMO samples were investigated by SEM, TEM and HRTEM as shown in Fig. 2. In Fig. 2a, the EG&M sample exhibits a foamlike surface, stacked with agglomerated particles with high porosity. The catalyst shows a macroporous surface structure with aperture size above 50 nm,^38,39^ and they are ineffective in increasing specific surface area (SSA) as indicated in Table 1. These features are beneficial for exposing Pd nanoparticles as active sites and improving metal dispersion.^27^Fig. 2b shows the morphology of the CP sample where agglomerated nanoparticles with uniform size are formed. Interestingly, the supported samples maintain their initial morphologies of pristine SMO samples (Fig. S3†). In TEM images of Fig. 2c and d, one can observe that the particle size of the catalysts was 50–60 nm, approximately four times larger than the value (13 nm) estimated from XRD analysis (Table 1). The pore size and volume tends to decrease as shown in Table 1, implying that the pore was partially occupied by the Pd species after the impregnation procedure.^40^ Thus, the larger pore size is beneficial to exposing superficial active site of Pd particles. As expected, the CO chemisorption analysis (Fig. S4†) illustrates a higher dispersion of Pd for the EG&M sample (38.4%) compared with that of the SMO-CP sample (16.6%). Using the eqn S(3),† the metallic Pd particle size was in the ranges of 2.7–3.0 nm for Pd/SMO-EG&M and 5.9–7.5 nm for Pd/SMO-CP, respectively*.* This suggests the suppression of Pd aggregation on SMO-EG&M support.^26,38^ In HRTEM images of Fig. 2e and f, the PdO_*x*_ particles are clearly observed and segregated on the mullite surface with a mean particle size of 3 nm and 6 nm for EG&M and CP samples, which is well consistent with the estimated value. The observed inter-planar distance of the particles in each figure is 0.223 and 0.254 nm approximately, corresponding to the (2 0 0) and (1 1 0) plane of tetragonal PdO_2_.^41,42^ In addition, the presence of well-crystallized mullite-type SmMn_2_O_5_ is confirmed by its typical *d*-spacing (*d*_2 1 0_ = 0.293 nm and *d*_1 1 2_ = 0.253 nm).^23,28^

**Table 1:** Chemical composition and physico-chemical properties of the catalysts

Catalysts	Pd^a^ (wt %)	*D*_ave_ b (nm)	SSA^c^ (m^2^ g^−1^)	Pore size_ave_ (nm)	Pore volume^d^ (cm^3^ g^−1^)	Surface coverage^e^ (μmol g_cat_^−1^)	Pd dispersion^e^ (%)	Pd size^e^ (nm)
SMO-EG&M	—	12.56	16	41.6	0.11	—	—	—
SMO-CP	—	13.26	15	15.1	0.05	—	—	—
Pd/SMO-EG&M	0.51	13.04	12	37.2	0.08	18.0 ± 1.0	38.4 ± 2.2	2.7–3.0
Pd/SMO-CP	0.53	13.01	10	14.6	0.04	7.9 ± 0.9	16.6 ± 2.0	5.9–7.5

a ICP-OES.

b Debyb–Scherrer formula.

c BET method.

d *t*-Plot method.

e CO-chemisorption.

### 3.2. XPS

**Fig. 3:**
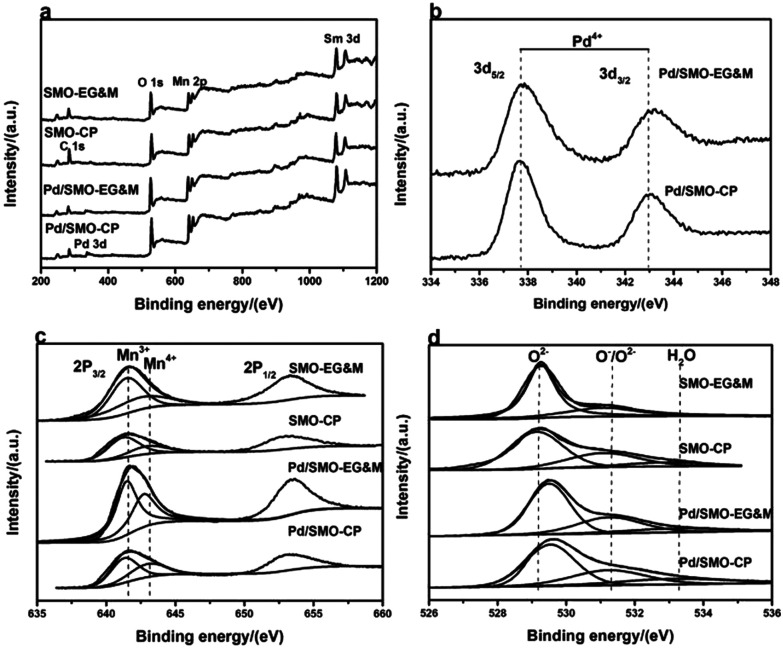
XPS spectrum of SMO and Pd/SMO: (a) survey spectrum; (b) Pd 3d; (c) Mn 2p; (d) O 1s.

The surface composition and chemical electronic states of the samples was examined by XPS. High resolution XPS spectrum of Pd 3d is included in Fig. 3b. Briefly, the components Pd 3d_5/2_ at 337.7 eV and Pd 3d_3/2_ at 342.7 eV are observed, herein the BE of the Pd 3d_5/2_ is higher than that of Pd^2+^ ion (336.1–336.9 eV), indicating the formation of highly oxidized cationic of palladium species (Pd^3+^ or Pd^4+^).^19,43–45^ This behavior is in good agreement with the FTEM image. Recent reports have shown that the higher oxidized Pd species, such as PdO_2_, displays a high thermal stability and reactivity toward CO oxidation at room temperature,^42,46,47^ thus we speculate that the performance of CO oxidation for mullite could be enhanced after Pd addition. Fig. 3c illustrates the Mn 2p XPS spectrum with Mn 2p_3/2_ at 641.9 eV and Mn 2p_1/2_ at 652.9 eV, respectively. Herein, the lower asymmetrical Mn 2p_3/2_ peak can be fitted into two components with a BE of 641.3–641.7 eV and 642.2–643.0 eV, referring to the Mn^3+^ and Mn^4+^ species, respectively.^24,27–29^ The surface Mn^4+^/Mn^3+^ ratio of 0.72 deviates from the theoretical value of 1.0 (Table S2†), which suggests the surface enrichment of Mn^3+^.

Fig. 3d is the fined scanned XPS spectrum of the O 1s. Three types of signals at low BE (529.3–529.9 eV), medium BE (531.0–531.4 eV) and high BE (above 533.2 eV) are associated with lattice oxygen (O_latt_: O^2−^), surface adsorbed oxygen (O_ads_: O^2−^, O^−^ or OH groups) and adsorbed molecular water, respectively.^26–28,48^ For the pristine EG&M sample, the BE values for lattice oxygen and surface adsorbed oxygen species are 529.1 eV and 531.2 eV, respectively. However, the values shift to 529.7 eV and 531.4 eV after Pd addition, and a similar behavior is found for the CP sample. Induced by defects of palladium oxide, the presence of a larger amount of oxygen vacancies is demonstrated by the increase of BE, which illustrates higher capacity of electronic capture and release, further accelerating the mobility and redox properties of reactive oxygen species.^28,49^ Consistent with our previous work,^27^ the SMO-EG&M sample possesses a larger O_ads_/O_latt_ ratio than that of SMO-CP (Table S2†). After Pd addition, the values increase to 0.54 and 0.49 for Pd/SMO-EG&M and Pd/SMO-CP, respectively. The larger O_ads_ concentration for the supported samples is attributed to the well dispersed Pd species, which facilitates the adsorption of dissociative oxygen in light of the oxygen spillover effect.^43–46^ Moreover, considerable interface would be formed between mullite and Pd species, with the formation of defect and oxygen vacancies, which explains the increased oxygen binding energy and O_ads_ concentration. Since a higher O_ads_ concentration is beneficial for oxidative reaction, the Pd/SMO-EG&M exhibits higher catalytic activity. Besides, the reducibility and mobility of O_ads_ are crucial for CO and C_3_H_8_ oxidation as O_ads_ should be activated at the first step,^47,49^ which will be investigated by the following O_2_-TPD and H_2_-TPR measurements.

### 3.3. O_2_-TPD and H_2_-TPR

**Fig. 4:**
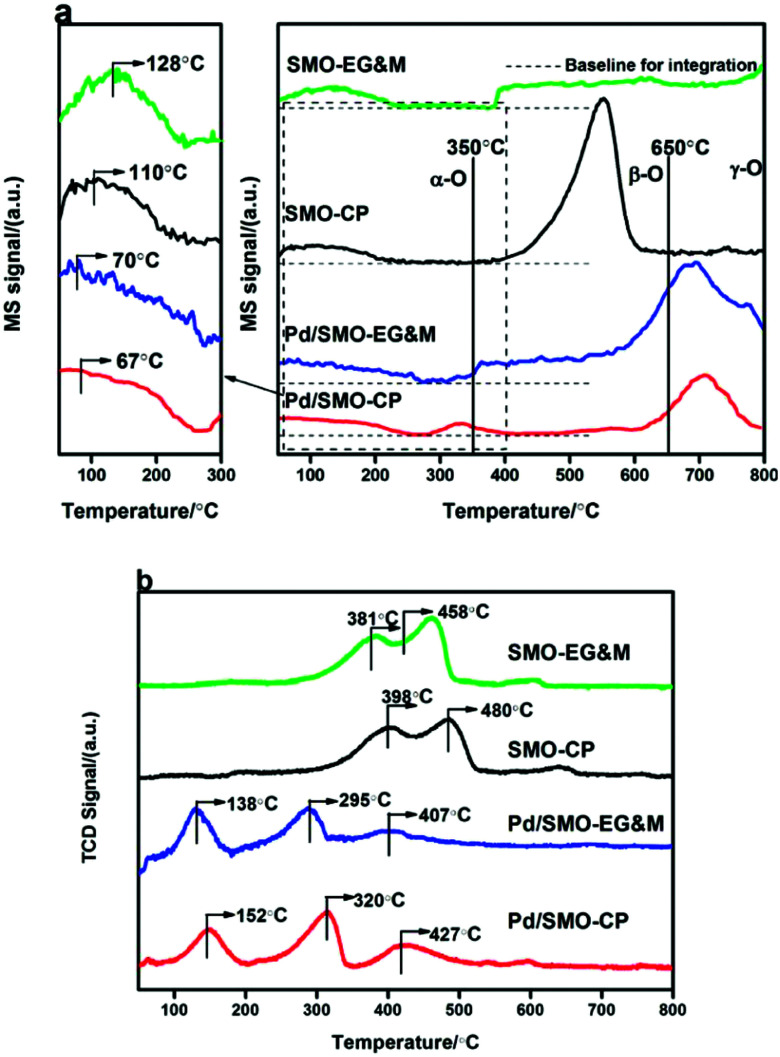
(a) O_2_-TPD and (b) H_2_-TPR profiles of the SMO and Pd/SMO.

The oxygen desorption behavior for the prepared samples was investigated by O_2_-TPD as shown in Fig. 4a. The amount of desorbed oxygen was estimated and listed in Table 2. Overall, the desorption profile is categorized into three regions with the demarcation intervals of 350 °C and 650 °C, where the desorbed oxygen species are denoted as α-O, β-O and γ-O, respectively.^27^ α-O is known to represent the weakly bonded oxygen species on the surface (O^2−^ and O^−^ species), which is responsible for oxidative reactions at relatively low temperature.^50–54^ β-O and γ-O, showing correlated response to methane oxidation, are ascribed as the release of equivalent oxygen in surface lattice and bulk mullite skeleton, respectively.^27,51,53–56^ Note that the temperature window of CO and C_3_H_8_ oxidation was mainly encompassed with α-O, its concentration and mobility would largely affect the catalytic performance. In the enlarged figure of the desorption region, we see that the signal of α-O shows a weak plateau-like peak with its concentration in the order of Pd/SMO-EG&M (38.5 μmol g^−1^) > Pd/SMO-CP (27.9 μmol g^−1^) > SMO-EG&M (24.9 μmol g^−1^) > SMO-CP (20.4 μmol g^−1^), speculating that the generation of surface oxygen species is more facile for the Pd/SMO-EG&M, which is well correlated with the highest ratio of O_ads_/O_latt_ for the Pd/SMO-EG&M as attained from XPS results. The higher concentration of α-O for Pd/SMO-EG&M is attributed to the stronger oxygen spillover effect between Pd species and EG&M support (Fig. S6†). In addition, the higher mobility of active oxygen species are crucial for CO oxidation and suggested by the lower desorption temperature. We see the desorption temperatures of α-O follow the sequence of Pd/SMO-CP (67 °C) ≈ Pd/SMO-EG&M (70 °C) < SMO-EG&M (110 °C) < SMO-CP (128 °C), suggesting the stronger mobility of O_ads_ after Pd addition. The high mobility indicates an ease of oxygen migration, which provides sufficient available oxygen species and is beneficial for activation of reactants.

**Table 2:** The quantitative amount analysis result of TPD profiles

Catalysts	O_2_ desorption amount^a^ (μmol g^−1^)			CO adsorption amount^b^ (μmol g^−1^)		CO_2_ adsorption amount^c^ (μmol g^−1^)	
	α-O (<350 °C)	β-O (350–650 °C)	γ-O (>650 °C)	<350 °C	350–800 °C	<450 °C	450–800 °C
SMO-EG&M	24.9	59.1	33.8	100.5	139.6	42.6	7.1
SMO-CP	20.4	135.2	0.7	83.7	81.0	39.9	2.7
Pd/SMO-EG&M	38.5	75.8	97.4	151.7	145.6	60.6	13.5
Pd/SMO-CP	27.9	13.6	51.0	129.7	231.6	50.3	13.6

a Desorbed concentration of O_2_ during O-TPD (Fig. 4).

b Desorbed concentration of CO_2_ during CO-TPD (Fig. S5).

c Desorbed concentration of CO_2_ during CO_2_-TPD (Fig. S6).

The reduction behavior of the catalysts was studied by H_2_-TPR. As shown Fig. 4b, the TPR profiles of the pristine EG&M sample present three reduction peaks, which indicate the multiple reductive processes for SMO mullite. The first two consecutive peaks at 381 °C and 458 °C result from the consumption of adsorbed oxygen and reduction of Mn^4+^ to Mn^3+^.^24,27,28^ After the deposition of Pd, the release of the surface oxygen species for EG&M sample is greatly promoted as evidenced by the decline of reduction peak to 138 °C. In addition, the reduction temperature of Mn^4+^ and Mn^3+^ decreases to 295 °C and 407 °C, suggesting the facile reduction of adsorbed oxygen and Mn ion by loading palladium species. The reason for the decrease of reduction temperatures is attributed to the hydrogen spillover effect, because hydrogen is easily dissociated at the reduced Pd surface and spilled to the SMO support.^57–59^ In addition, the enhanced activity of oxygen species (certified by CO + O_2_ pulse hereafter) contributes as well.^28,37^ As the Pd dispersion is higher for the Pd/SMO-EG&M compared with other samples, its surface Pd site was highly exposed and could be reduced easier. The lower temperature suggests higher reducibility of the Pd/SMO-EG&M in the whole range, suggesting that the reduction of mobile oxygen and surface adsorbed oxygen species is more facile. The superior reducibility for the Pd/SMO-EG&M indicates a stronger Pd-interaction, and it is beneficial for exhaust removal.

### 3.4. CO + O_2_ pulse test

**Fig. 5:**
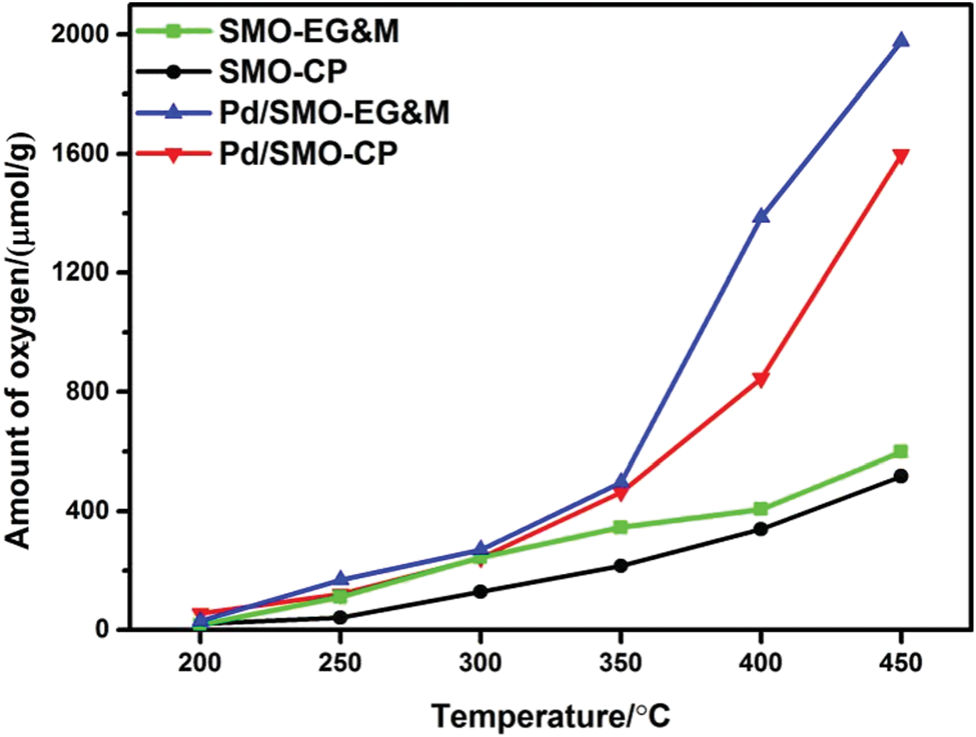
OSC of the SMO and Pd/SMO.

The oxygen storage capacity (OSC) has been commonly applied as a critical factor to quantify the amount of active oxygen available for chemical reactions. Following the thermodynamic and kinetic laws, the diffusion of lattice oxygen is largely accelerated with rised temperatures, which enhances the release of oxygen for all samples.^24,60–62^ In Fig. 5, we see that all samples show similar OSC below 250 °C, while the desorbed oxygen species that include motivated surface adsorbed oxygen and lattice oxygen increase rapidly above 250 °C for the supporting samples. Based on CO-TPR profile (Fig. S6†), we find that the reduction of adsorbed oxygen and highly oxidized manganese ion occurs in this region, which leads to higher CO consumption in reductive pulse. In oxidation pulse, the Pd species could accomplish a quick oxygen migration and accelerate the reoxidation of reduced manganese ion *via* the oxygen spillover effect (S10†). Hence, better redox capacity of oxygen species and manganese ion lead to higher CO_2_ production for all supported samples. Moreover, we see that Pd/SMO-EG&M shows a lower reduction temperature compared with the Pd/SMO-CP from the CO-TPR (Fig. S6†). Indeed, the Pd/SMO-EG&M sample possesses a higher amount of activated oxygen species than that of the Pd/SMO-CP in the whole temperature range, which is in agreement with the results of O_2_-TPD and CO_2_-TPD (Fig. S6†). The origin of easier activation of O_2_ is attributed to a larger amount of adsorption sites on EG&M support, which contributes to easier accessibility of active sites and activation of reactants (CO, C_3_H_8_ and O_2_). Note that the oxidation temperature for C_3_H_8_ exceeds 250 °C, we conclude that Pd/SMO-EG&M provides sufficient active oxygen species and displays the superior catalytic performance.

### 3.5. Performance evaluation

**Fig. 6:**
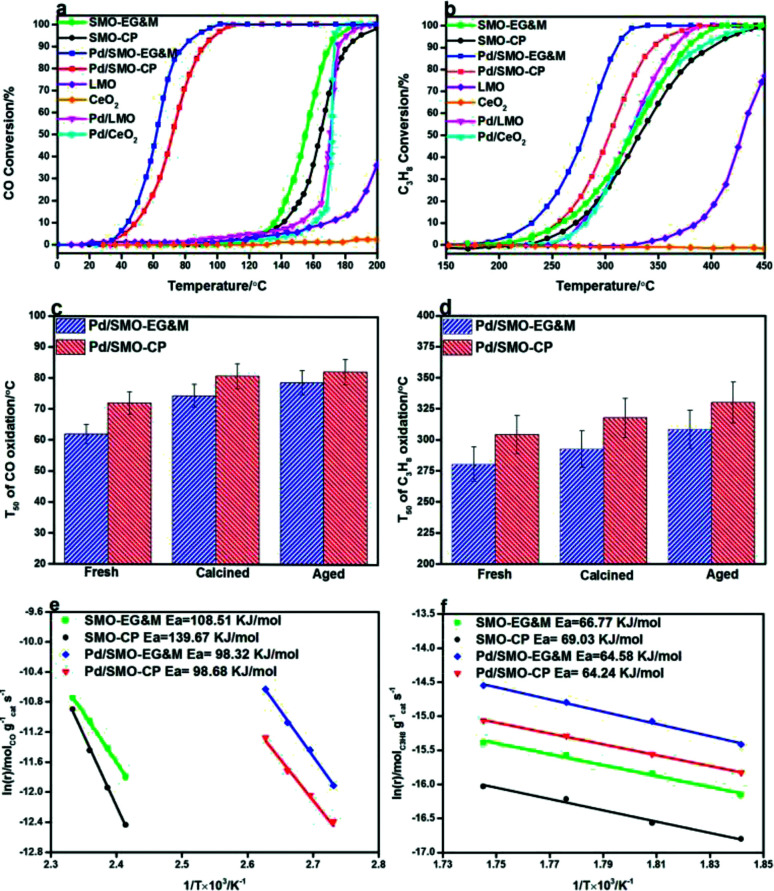
(a) Catalytic activities of CO oxidation. Feed: 1% CO, 10% O_2_ and N_2_ as balance; (b) catalytic activities of C_3_H_8_ oxidation. Feed: 500 ppm C_3_H_8_, 10% O_2_ and N_2_ as balance; (c) *T*_50_ of CO oxidation curve catalyzed by Pd supported catalysts after calcination of hydrothermal aging treatment; (d) *T*_50_ of C_3_H_8_ oxidation curve catalyzed by Pd supported catalysts after calcination of hydrothermal aging treatment; (e) Arrhenius plots for CO oxidation; (f) Arrhenius plots for C_3_H_8_ oxidation.

The combustion activity of CO and C_3_H_8_ for were given in Fig. 6. In panel (a), the SMO-EG&M achieves a significant conversion of CO at 160 °C, comparable to that of supported Pd/CeO_2_ and Pd/LMO conventional catalysts. Through deposition of 0.5 wt% Pd over the catalyst, the corresponding temperatures of light-off curve drop by 75 °C relative to the pristine SMO-EG&M sample. Similarly, considerable catalytic performance is identified below 250 °C and complete conversion of C_3_H_8_ is obtained at 320 °C after Pd addition, as seen in Fig. 6b. The similar improved catalytic efficiency is observed for CP sample as well, whereas its combustion activity is lower compared with EG&M. All Pd/SMO catalysts display better catalytic performance than the SMPO reference catalysts (Fig. S8†). In addition, we extensively compared the catalytic activity of Pd/SMO with some other Pd supported catalysts reported in literature, the results were listed in Table 3. It is found that the Pd/SMO-EG&M catalyst exhibits an optimal catalytic activity for CO and C_3_H_8_ oxidation with lower *T*_50_ values, lower Pd loading and higher gas hourly space velocity. The CeO_2_–Co_3_O_4_ and CeO_2_–CuO catalysts have shown better C_3_H_8_ oxidation catalytic activity, mainly due to the lower gas hourly space velocity and higher BET surface area.

**Table 3:** Comparative assessment for activity of various Pd catalysts for CO or C_3_H_8_ oxidation

Supports	Pd content (wt%)	*T*_50_ (°C)		Reaction conditions	Ref.
		CO	C_3_H_8_		
SMO-EG&M	0.5	61.9	280.2	1% CO/500 ppm C_3_H_8_, 10% O_2_, N_2_ balance, 150 mL min^−1^, 50 mg	This work
SMO-CP		71.9	304.1		
CeO_2_–Co_3_O_4_	0.5	70.0	231.4	1% CO/0.5% C_3_H_8_, 5% O_2_, N_2_ balance, WHSV = 15 000 mL g^−1^ h^−1^, 600 mg	1
CeO_2_–NiO_*x*_		99.0	298.4		
CeO_2_–MnO_*x*_		<40.0	240.6		
CeO_2_–CuO		66.0	289.7		
CeO_2_–R	1.0	—	401.0	1% CO, 20% O_2_, Ar balance, 50 mL min^−1^, 10 mg/0.2% C_3_H_8_, 2% O_2_, Ar balance, 100 mL min^−1^, 20 mg	63
CeO_2_–C		—	336.0		
CeO_2_–O		—	268.1		
LaCoO_3_	2.1	210.1	352.1	6% CO, 0.2% C_3_H_8_, 4.4% O_2_, Ar balance, 50 cm^3^ STP per min, 25 mg	64
LaCoO_3_-reduced		162.3	303.5		
La_2_O_3_	1.0	210.1	—	1% CO, 21% O_2_, N_2_ balance, 30 mL min^−1^, WHSV = 72 000 mL g^−1^ h^−1^	65
SnO_2_		162.3	—		
Al_2_O_3_		157.2	—		
CeZr/Al_2_O_3_	2.0	—	348.2	0.3% C_3_H_8_, 3% O_2_, N_2_ balance, GHSV = 30 000 h^−1^, 600 mg	66
CeZr–Y/Al_2_O_3_		—	312.8		
ZSM-5	1.5	—	327.1	2000 ppm C_3_H_8_, 2% O_2_, N_2_ balance, 100 mL min^−1^, 200 mg	67

A high temperature of 900 °C is usually encountered in automobile exhaust where catalysts are easily sintered and deactivated.^68^ To examine the industrial applications, the resistance of thermal and hydrothermal aging of the samples were measured. The *T*_50_ of CO and C_3_H_8_ oxidation is plotted in Fig. 6c and d, respectively. Despite a severe agglomeration of Pd and SMO support (Fig. S13 and S14†), both samples maintain their good CO oxidation activity after calcination or aging treatment. For C_3_H_8_ oxidation, the Pd/SMO-EG&M sample still shows a *T*_50_ below 320 °C, which indicates most of active sites are maintained under harsh conditions. The observations demonstrate the Pd/SMO-EG&M sample with higher Pd dispersion exhibits the higher activity and stability, with great promise in purification of hazardous emissions.

Arrhenius plots of ln *r versus* 1/*T* for CO and C_3_H_8_ oxidation are displayed in Fig. 6e and f, respectively. For CO oxidation dynamic test, we can see that the reaction rate of EG&M sample is higher compared to that of CP sample at the whole reaction region in Fig. 6e, which is consistent with the result of catalytic performance results. The apparent activation energy (*E*_a_) for all samples was calculated and ranked in the order SMO-CP (139.67 kJ mol^−1^) > SMO-EG&M (109.51 kJ mol^−1^) > Pd/SMO-CP (98.68 kJ mol^−1^) ≈ Pd/SMO-EG&M (98.32 kJ mol^−1^). In Fig. 6f, a similar sequence of reaction rate and *E*_a_ are observed for C_3_H_8_ oxidation. The higher reaction rate and lower activation energy for Pd/SMO-EG&M indicate that CO and C_3_H_8_ oxidation proceed more easily with macroporous SMO support. The Pd supported catalysts show similar TOF_Pd_ values for CO oxidation at 100 °C and for C_3_H_8_ oxidation at 300 °C (Table S3†), suggesting an identical active center for CO and C_3_H_8_ oxidation. Normally, TOF_Pd_ represents the converted number of the reactant on per Pd site. Thus, with an identical TOF_Pd_ value, a larger number of active sites over EG&M support would give rise to the higher reaction rate and activity.

**Scheme 1:**
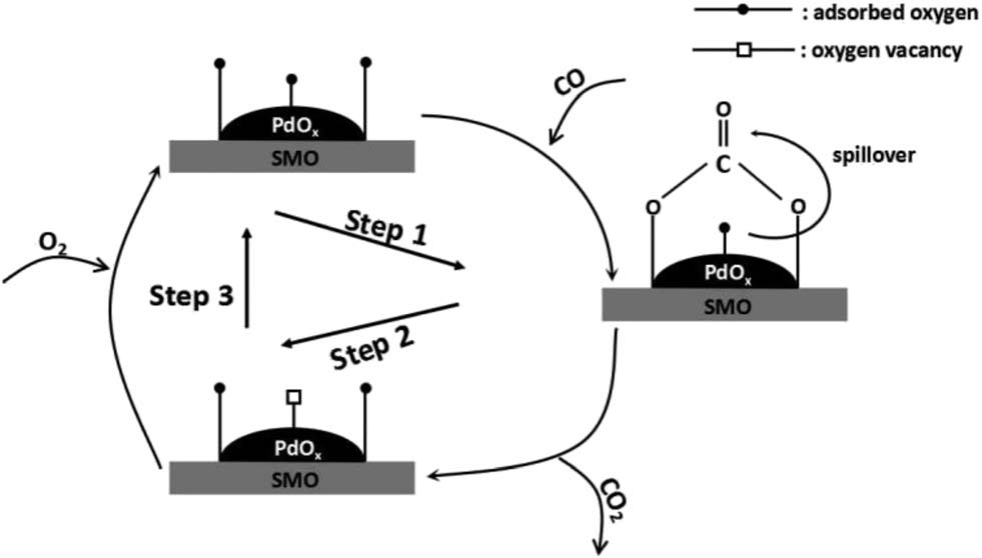
The reaction pathway for CO oxidation of the Pd/SMO catalysts.

Scheme 1 illustrates the detailed reaction pathway of Pd/SMO catalysts for CO oxidation. In brief, CO molecule is chemisorbed on the surface of the catalyst (Step 1) and reacts with surface active oxygen species to form bidentate carbonate, followed by its decomposition into CO_2_ (Step 2) to regenerate oxygen vacancy.^1,37^ Finally, gas-phase oxygen is replenished onto the oxygen vacancies to accomplish the redox cycle (Step 3). During the whole procedure, the activation of surface oxygen species are supposed to play a rate-determination step since the reaction occurs at low temperatures where little oxygen species are motivated in this temperature range as illustrated in OSC test. Thus, fast oxygen activation plays a critical role in CO oxidation. H_2_-TPR test has revealed the easily reducible surface O_ads_ for the Pd/SMO-EG&M, and its mobility is higher by the stronger spillover effect as demonstrated in TPO test. Most of active sites for CO oxidation are at the interface between transition metal oxide and the supported species where spilled oxygen are transmitted *via* support.^1,36,37^ In this way, the oxygen species of PdO_*x*_ on EG&M support could be motivated at lower temperatures and spilled to the surface of catalyst, and such activated oxygen atom could preferentially react with the adsorbed CO and form bidentate carbonate *via* spillover, giving rise to the improved catalytic performance. Moreover, due to a larger number of exposed Pd sites, the Pd/SMO-EG&M is beneficial to CO chemisorption (Fig. S5†), which leads to the improved CO oxidation activity as well.

When it comes to C_3_H_8_ oxidation, the oxygen activation is largely accelerated in this temperature region, the fast oxygen activation is no longer regarded as the rate-limiting step. Note that the light-off temperature for the saturated conversion is closely associated with the strength of C–H bond, the reaction rate is decided by the abstraction of H atom in C_3_H_8_ molecule.^1,58,59^ After abstracting C–H bond, the active oxygen species available for hydrocarbons oxidation becomes a dominate factor.^1,69^ A larger number of basic centers of Pd/SMO-EG&M has been confirmed by CO_2_-TPD measurement (Fig. S5†) and demonstrates an easier activation of C–H bond. The O_2_-TPD and CO + O_2_ pulse tests display higher mobility and OSC for Pd/SMO-EG&M above 250 °C, confirming higher amount of available oxygen species for C_3_H_8_ activation. Above all, the higher dispersion of Pd species on EG&M support suggests the presence of larger amount of Pd active sites,^26,38^ leading to stronger synergistic effect and serving superior catalytic performance of CO and C_3_H_8_ oxidation. In a word, this catalyst has great potential in exhaust purification for gasoline vehicle for it shows a high catalytic efficiency at low temperatures even after calcination of hydrothermal aging treatment for a long time.

## 4. Conclusions

Pd-decorated SMO mullite catalysts have been synthesized *via* an incipient-wetness impregnation method. The Pd/SMO-EG&M sample with excellent CO and C_3_H_8_ oxidation catalytic activity possesses a higher dispersion and smaller particle size of Pd species than that of CP sample. The higher Pd dispersion on EG&M support is beneficial to exposing active sites and enhancing the reducibility of O_ads_, which accelerates the oxygen spillover effect and promotes its CO oxidation capability. The higher oxygen mobility for Pd/SMO-EG&M means a facile oxygen transfer and helps the activation of available oxygen species above 250 °C, improving the activation of C–H bond and further resulting in optical C_3_H_8_ oxidation performance. It is concluded that SMO support functions well with outstanding thermal and structural stability, which exhibits great potential to ravel out the existing problems associated with metal oxide/Pd catalysts.

## Conflicts of interest

The authors declare that there are no conflicts to declare.

## Supplementary Material

RA-008-C7RA11551B-s001
