# Supplementary material for: Highly dispersed Pd on macroporous SmMn2O5 mullite for low temperature oxidation of CO and C3H8
Source: RSC Adv. 2018 Jan 31;8(10):5459–67. doi: 10.1039/c7ra11551b (PMC9078158; doi:10.1039/c7ra11551b)
Supplement: RA-008-C7RA11551B-s001 [file RA-008-C7RA11551B-s001.pdf]

Electronic Supplementary Material (ESI) for RSC Advances.
This journal is © The Royal Society of Chemistry 2018

Highly dispersed Pd on macroporous $SmMn_2O_5$ mullite for low temperature oxidation of CO and C_3H_8

Yuning Zhu,‡[a] Chun Du,‡[a] Zijian Feng,[a] Yongjie Chen,[a] Hang Li,[c] Rong Chen,[b] Meiqing Shen[c] and Bin Shan*[a]

[a] State Key Laboratory of Material Processing and Die and Mould Technology and School of Materials Science and Technology, Huazhong University of Science and Technology, Wuhan 430074, Hubei, China.
[b] State Key Laboratory of Digital Manufacturing Equipment and Technology and School of Mechanical Science and Engineering, Huazhong University of Science and Technology, Wuhan 430074, Hubei, China.
[c] School of Chemical & Engineering and Technology, Tianjin University, 92 Weijin Road, Nankai District, Tianjin 300072, China.

‡These authors contributed equally to this work.

* Corresponding author: Bin Shan
Postal address:
State Key Laboratory of Material Processing and Die and Mould Technology and School of Materials Science and Technology, Huazhong University of Science and Technology, Wuhan 430074, Hubei, People’s Republic of China.
E-mail address: bshan@mail.hust.edu.cn.

S1. Preparation of $SmMn_2O_5$ and Pd/$SmMn_2O_5$ catalysts

Macroporous $SmMn_2O_5$ mullite powder was synthesized using a combustion of ethylene glycol and methanol solution method (denoted as SMO-EG&M). In a typical synthesis, stoichiometric amounts of $Sm(NO_3)_3·6H_2O$ and $Mn(C_2H_3O_2)_2·4H_2O$ were dissolved in an aqueous solution containing 6 mL ethylene glycol and 4 mL methanol. The solution was stirred constantly to achieve a uniform precursor. After dehydration at room temperature overnight, the final EG&M powders were obtained by combusting organic solution at 500 °C and 800 °C for 8 h in air, respectively. For comparison purposes, the conventional bulk $SmMn_2O_5$ was prepared by a conventional co-precipitation method (denoted as SMO-CP). Briefly, stoichiometric $Sm(NO_3)_3·6H_2O$ and $Mn(C_2H_3O_2)_2·4H_2O$ were dissolved in 40 mL of deionized water under adequate stirring with the addition of appropriate Pluronic F127. After Defoaming with octanol, the homogeneous solution was precipitated with pH adjusted to 10.0 and oxidized in sequence with the dropwise addition of tetramethylammonium hydroxide (TMAH, 25 wt% in water) and hydrogen peroxide (H_2O_2, 30 wt% in water), respectively. After 2 hours aging, the obtained mixture was then centrifuged, filtered and dried at 120 °C whole night and subsequently calcined under the same conditions as SMO-EG&M.)

The Pd/SMO-EG&M and Pd/SMO-CP samples were then prepared by the incipient-wetness impregnation method. 1g SMO powders were immersed with an aqueous $Pd(NO_3)_2$ (18.09 wt% in water) solution and dried at 100 °C for 6 hours, followed by calcining at 500 °C for 2 hours. Meanwhile, to compare their catalytic property with commercial mostly used catalysts, 0.5 wt% Pd on CeO_2 and on $LaMnO_3$ were also prepared and denoted as Pd/CeO_2 and Pd/LMO, respectively. In this paper, all the chemicals and regents were analytical purity and purchased from the company of Sigma-Aldrich and Sinopharm Chemical Reagent Co., Ltd.

To evaluate their practical potential to the realistic vehicle ambience, the supporting samples were calcined in oxidative and moisture atmosphere, named thermal aging and hydrothermal aging treatment, respectively. In the former condition, the samples were placed in tube furnace and heated at 900 °C in air (1000 mL min^{-1}) for 2 hours, labeled as EG&M-calcined and CP-calcined, respectively; while in the latter conditions, the samples were heated for 10 hours under 10% H_2O with air as balance with the same flow rate and calcined temperature, labeled as EG&M-aged and CP-aged, respectively.

S2. Detailed experimental process

The temperature-programmed reduction (H_2-TPR and CO-TPR), desorption (O_2-TPD, CO-TPD and CO_2-TPD) and oxidation (TPO) measurements were undertaken on an automatic adsorption instrument (FINESORB-3010E, Zhejiang Finetec Co). Prior to the H_2-TPR test, 50 mg of the catalyst was firstly activated with 100 mL/min flow of 10% O_2/N_2 at 500 °C for 30 minutes. After cooling down to room temperature, 50 mL/min of 10% H_2/Ar was switched into the system with the signal of H_2 concentration recorded by a thermal conductivity detector (TCD). Afterwards, the sample was heated up to 800 °C at 5 °C min^{-1}. By replacing 10% H_2/Ar with 1% CO/Ar as a reducing agent, the CO-TPR measurement was also carried out. For TPD measurement, 100 mg of sample was activated as aforementioned conditions and cooled to room temperature. After swept with pure He, the samples were saturated with O_2, CO and CO_2 for 30 min, respectively. Following completion of the adsorption, the sample was swept by 70 mL/min of He at room temperature to remove the residual gases of the system. After 3 hours stabling, the temperature was then raised to 800 °C at a rate of 10 °C/min. In the TPO test, 50 mg powder was pre-reduced under 10% H_2/Ar flow at 700 °C and held for 30 min to accomplish a complete reduction, followed by cooled to room temperature. Afterwards, the powder was fed with atmosphere of 0.5% O_2/Ar mixture with total flow rate of 100 mL/min and then heated to 800 °C at a ramp rate of 10 °C/min. For TPD and TPO, the gases in the outlet were collected by a quadrupole mass spectrometer (Hiden HPR-20), and the evolution of O_2, CO and CO_2 during each procedure was recorded with its m/z value equaled to 32, 28 and 44, respectively. Prior to the measurements, the calibration of background and each component were conducted, and the fragment of CO_2 at 28 was eliminated.

The oxygen storage capacity (OSC) was measured on the same apparatus to reveal the reactivity of surface and bulk oxygen species. Briefly, 25 mg of catalyst was diluted with 45 mg quartz particles and exposed to a sequence of oxidizing (4% CO/He) and reducing (2% O_2/He) pulse in each given temperature. For each pulse, the duration was set as 5s with the concentration of CO, O_2 and CO_2 in the outlet gas flow quantified by mass spectrometer (Hiden HPR-20). The testing temperature ranged from 200 °C to 450 °C with a step of 50 °C. A dynamic OSC value was

estimated from the the consumption of O_2 in each cycle, which could be evaluated via integrating the CO_2 concentration versus time response curve with one CO-O_2 cycle.

The oxygen storage capacity (OSC) was measured on the same apparatus to reveal the reactivity of surface and bulk oxygen species. Briefly, 25 mg of catalyst was diluted with 45 mg quartz particles and exposed to a sequence of oxidizing (4% CO/He) and reducing (2% O_2/He) pulse in each given temperature. For each pulse, the duration was set as 5s with the concentration of CO, O_2 and CO_2 in the outlet gas flow quantified by mass spectrometer (Hiden HPR-20). The testing temperature ranged from 200 °C to 450 °C with a step of 50 °C. A dynamic OSC value was estimated from the the consumption of O_2 in each cycle, which could be evaluated via integrating the CO_2 concentration versus time response curve with one CO-O_2 cycle.

The catalytic activity tests were performed on FINESORB-3010E. 50 mg of the catalyst was packed in a U-type vertical quartz tubular reactor (i. d. = 5 mm). The total flow rate was set as 100 mL/min, corresponding to a gas hour space velocity (GHSV) of 120, 000 mL g^{-1} h^{-1}. The feed gas mixture, consisting of 1% CO/(10% O_2 + He) or 500 ppm C_3H_8/(10% O_2 + He) was flowed through the reactor. The sample was heated to 200 °C and 450 °C at the rate of 2 °C/min and 4 °C/min after 30 min stabilizing for CO and C_3H_8 oxidation test, respectively. The outlet gases were measured and quantified using Hiden HPR-20. The conversion of gas X_{con} (%) (X=CO or C_3H_8) was defined as the percentage of X feed that has been reacted, and it was calculated using the following Eq. S(1):

$$X_{\text{con}}(\%) = \frac{X_{in} - X_{out}}{X_{in}} \times 100\% \quad (1)$$

where X_{in} and X_{out} denoted the volumetric concentration of X feed in the inlet and outlet gas, respectively. T_{10}, T_{50} and T_{90} were corresponded to the temperature of 10%, 50% and 90% conversion, respectively.

S3. Preparation and XRD spectra of Pd/CeO_2 and Pd/LCO

The CeO_2 nanorod was prepared by the conventional hydrothermal method without template[1]. Briefly, appropriate amounts $Ce(NO_3)_2{\cdot}6H_2O$ and NaOH were dissovled in 35 ml of deonized water and constantly stirred for 30 min. Then, the milky slurry was transferred into stainless steel Teflon-lined autoclave and put in an oven at 100 °C and kept for 24 h. Afterwards, the final products were filtered and dried at 80 °C for 16 h. Finally, the obtained powder was ground and calcined at 500 °C for 4 h.

Sample LaMnO3 (LMO) was prepared by a citric acid sol–gel method[2]. Specifically, stoichiometric metal nitrates ($La(NO_3)_3{\cdot}6H_2O$, $Mn(NO_3)_2$ solution of 50

wt% in H_2O)) were dissolved in 60 ml deionized water. Then, citric acid was added into the solution with an equal citrate/total metal cations mol ratio. Subsquently, the solution was evaporated at 80 °C to obtain the viscous precursor. The obtained gel was dehydrated and dried at 90 °C overnight. The solid yellow foam was finely grinded and calcined under a static air atmosphere at 700 °C for 5 h to form the perovskite structure, respectively.

The XRD spectra of the Pd/CeO_2 and Pd/LMO sample were shown in Fig. S1. As expected, the single fluorite and hexagonal perovskite structures was obtained, respectively.

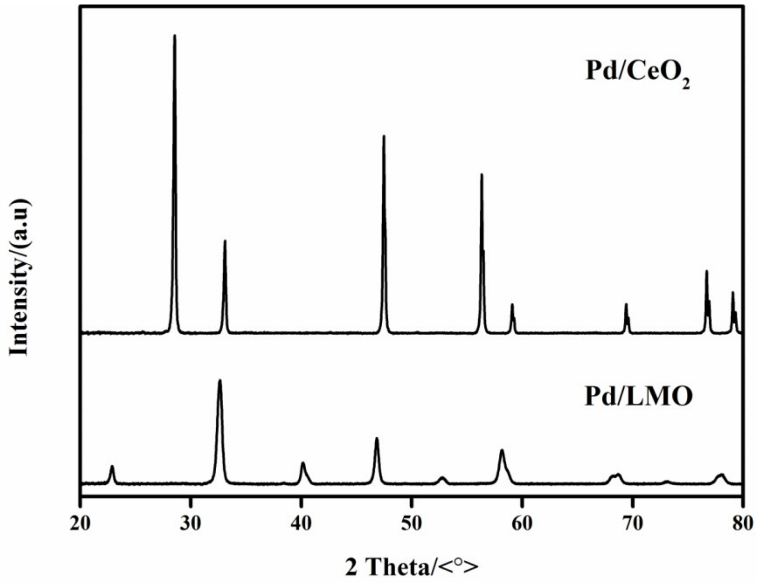

Fig. S1 XRD pattern of the Pd/CeO_2 and Pd/LMO sample.

S4. TEM image of Pd/CeO_2 nanorods

TEM was employed to examine the synthesis of CeO_2 nanorods as shown in Fig. S2. As observed, the CeO_2 nanorod is clearly observed and the Pd particles were well dispersed.

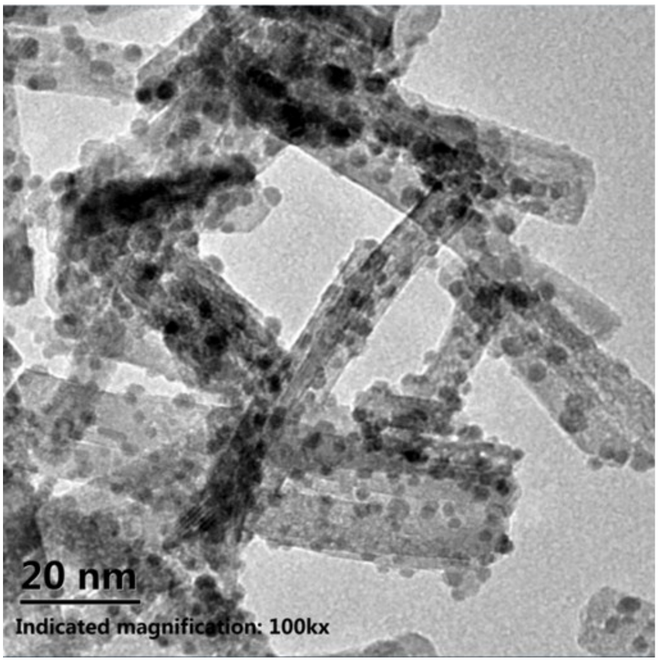

Fig. S2 TEM image of Pd/CeO_2 nanorod.

S5. Related calculation of crystalline size

The Full Width at Half Maximum (FWHM) values of X-ray diffraction were employed to calculate the crystalline size using Debyb-Scherrer formula in Eq. S(2) :

$$D = \frac{K\lambda}{\beta \cos\theta} \tag{2}$$

where K referred to the Scherrer constant, λ referred to the X-ray wavelength, β referred to the FWHM, and θ was the Bragg diffraction angle. With strongest XRD peak intensity, the (2 1 1) plane of SMO mullite at 30.6° was chosen as a basis of calculation. The relevant SMO crystalline parameters were listed below in Table S1, the mean grain size of SMO mullite was 13 nm.

Table S1 Relevant lattice parameters of (211) plane of mullite

	SMO-CP	SMO-EG&M	Pd/SMO-CP	Pd/SMO-EG&M
β of the (211) plane (°)	0.61	0.65	0.63	0.63
2θ of the (211) plane (°)	30.72	30.91	30.63	33.60

$d_{(211)}$ of mullite (Å)	2.91	2.89	2.92	2.93
D_{ave} (nm)	13.26	12.56	13.01	13.04

S6. SEM images of pristine mullite samples

The SEM images of pristine mullite are displayed shown in Fig. S3. Similar to the Pd supported catalyst, the SMO-EG&M and SMO-CP showed the morphology of agglomerate grape-like nanoparticles and macroporous structure, respectively. This result indicated that the structure of the support is retained after Pd addition.

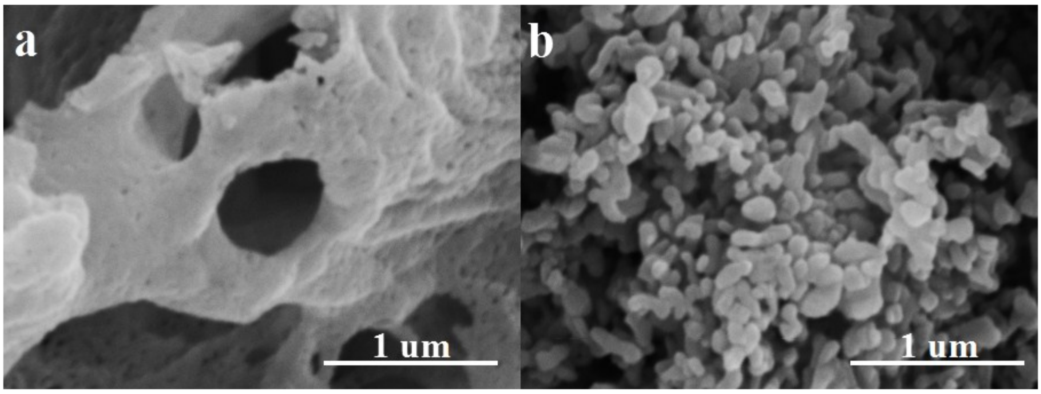

Fig. S3 SEM image of the: (a) SMO-EG&M; (b) SMO-CP sample.

S7. Pd dispersion determined by CO chemisorption

The dispersion of Pd species were determined using CO pulse chemisorption experiment. The CO adsorption uptakes was quantified using Hiden TPR-200. Prior to the measurement, 150 mg of the catalyst was preoxidized at 500 °C in air for an hour and reduced at 300 °C in a flow of 10% H_2/Ar for an hour, then a flow of pure argon at 400 °C was inleted to get rid of excess hydrogen. Subsequently, the pulse measurements were conducted by introducing 4%CO/Ar for 1 second in every 20 s at room temperature. As shown in Fig. S4, in each test, CO adsorption finished in first 3 pulses, so the CO uptake was calculated by subtracting the CO concentration in first 3 pulses from the steady amount of CO. In this study the dispersion was determined by assuming an equal adsorption stoichiometry of CO and Pd. The corresponding parameters were summarized in Table 1. Assuming the Pd particles to be spherical, the particle size can be determined by the value of dispersion using the following Eq. S(3):

$$d = \frac{6A}{\rho \sigma L D} \tag{3}$$

where A equals to the Pd atom mass (106.42 g·mol^{-1}), ρ equals to density (12.02×10^{6} g·m^{-3}) ,σ equals to average surface area occupied by one Pd atom (0.79×10^{-19} m^{2}) and L equals to the Avogadro's constant.

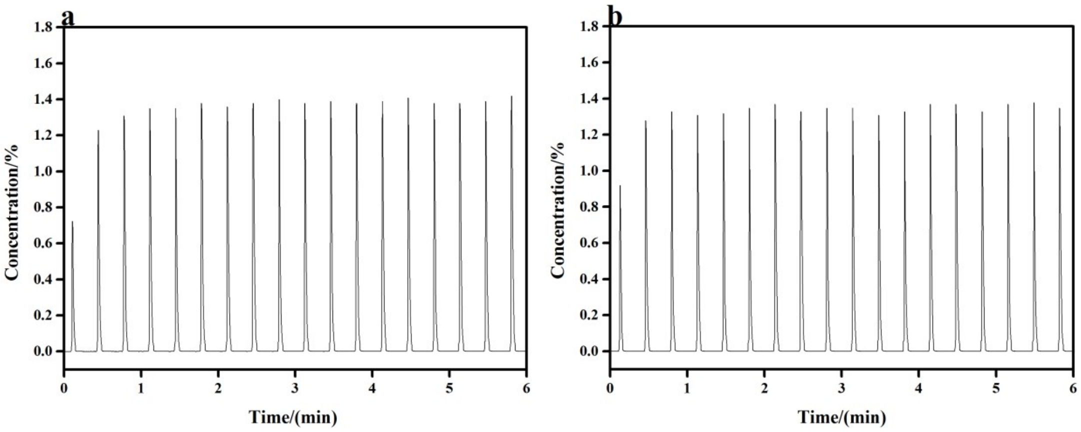

Fig. S4 CO signal in the pulse CO chemisorption experiment of the: (a) Pd/SMO-EG&M; (b) Pd/SMO-CP sample.

S8. Surface element ratio

Table S2 showed the surface atom ratio of the catalysts. The molar ratio of Sm/Mn and O/Mn is close to the theoretical value of 0.5 and 2.5 for all samples, whereas the the estimated Pd/Mn ratio is 40 times larger than the theoretical value (0.00014) because of the Pd surface enrichment.

Table S2 Surface element ratio of the catalysts

Catalysts	Sm/Mn	O/Mn	Pd/Mn	Mn^{4+}/Mn^{3+}	O_{ads}/O_{latt}
SMO-EG&M	0.60	2.86	-	0.74	0.42
SMO-CP	0.36	2.40	-	0.70	0.36
Pd/SMO-EG&M	0.39	3.32	0.095	0.75	0.54
Pd/SMO-CP	0.52	3.10	0.063	0.72	0.49

S9. Desorption of CO and CO_2

CO-TPD test was taken to explore CO adsorption of all catalysts, as shown in

Fig. S5a. Similar to previous report, there is no signal of CO and CO_2 is detected as desorbing gas for all samples[3-6]. For the pristine catalyst, a sharp CO_2 desorption peak appeared at 440 °C. Besides, we found an additional weak peak at 155 °C for EG&M sample. After Pd addition, both two catalysts display a major peak at 430 °C with a plateau-like shoulder below 300 °C, which confirmed the existence of carbonate species[5,6]. The amount of CO adsorption for EG&M increases from 100.5 μmol/g to 151.7 μmol/g after Pd addition (Table 2), thus we can conclude that the CO is easier to be oxidized to CO_2 on PdO_x than that on crystalline structure of mullite. Meanwhile, the higher capacity for CO adsorption at lower temperatures is found for the EG&M sample, which is attributed to higher Pd dispersion and stronger interaction on macroporous surface. The result indicates that the macroporous support was beneficial for exposing the active sites.

The CO_2-TPD measurement was conducted to characterize the surface basic strength. Generally, the basic strength of the catalysts were proposed as the ability of abstraction H atom from hydrocarbon molecule. It was known that desorption pattern at each given region and the desorption intensity indicated CO_2 adsorbed on different basic sites and the amount of basic sites, respectively[5-7]. In Fig. S5b, the desorption signal with varying intensities below 450 °C were clearly observed. However, the signal is absent at higher temperatures for all catalysts, which means that the weakly basic site is dominated on mullite surface. For the EG&M sample, the desorption peak at 112 °C evolved to a strong one at 71 °C with a shoulder at 204 °C after Pd addition. On the other hand, a larger number of basic sites were confirmed by the augmented intensity of the signal. Compared with the Pd/SMO-CP sample, the higher amount of desorbed CO_2 suggested that the Pd/SMO-EG&M possessed more active sites at low temperatures[5,7]. The larger amount of basic centers suggest higher amount of oxygen vacancies, accelerating the chemisorption of reactive oxygen species and activation of propane[3,5,7]. This was in agreement with the O_2-TPD results.

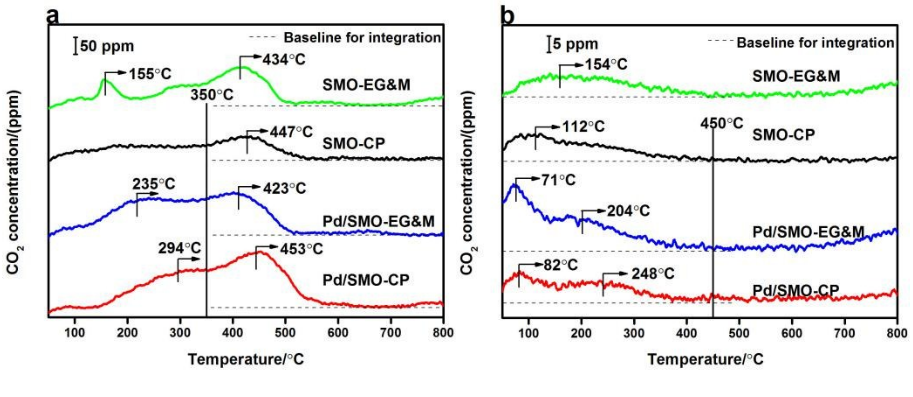

Fig. S5 (a) CO-TPD and (b) CO_2-TPD profiles of the SMO and Pd/SMO.

S10. Research of synergistic effect

Hydrogen spillover effect is commonly observed for supported catalysts. Once small amount of Pd oxide was reduced directly to metal Pd, the dissociation of H_2 molecules into reactive H atoms was triggered on Pd sites, which could diffuse to the

support and promote the subsequent reductive process[8,9]. To eliminate the spillover effect, CO-TPR experiment was conducted for all samples as CO was often molecularly chemisorbed on Pd site without dissociation. As shown in Fig. S6a, the reduction of SMO and Pd/SMO occurred in three steps as seen by two broad successive peaks and a narrow one, ascribed as the reduction of adsorbed oxygen, $Mn^{4+}O_x$ and $Mn^{3+}O_x$ in mullite structure, respectively. In contrast, the reduction procedure proceeded more rapidly for supported samples as evidenced by lower reduction temperature of adsorbed oxygen and lattice oxygen, indicating the higher oxygen mobility for the supported samples. This result was consistent with the result of O_2-TPD. Therefore, it could be concluded that both the spillover effect and the enhanced oxygen mobility could demonstrate the improved reducibility after Pd addition. For Pd supported samples, the Pd/SMO-EG&M sample showed lower reductive temperature, following the same trend as H_2-TPR. Consequently, concerning the results of H_2-TPR and CO-TPR, it was reasonable that both the spillover effect and the enhanced oxygen mobility could illustrate the improved reducibility. Such improvement was not observed in the Pd/Co_3O_4-CeO_2 sample[3,10,11], indicating the stronger interaction between mullite and Pd species.

The oxygen spilled effect was studied by the TPO test, as indicated in Fig. S6b. Note that the samples had been reduced at 700 °C, the manganese would be reduced as Mn^{2+} considering the result of H_2-TPR. Therefore, the consumption profile mainly includes the oxidation process of Mn^{2+} species. Briefly, there were two oxygen consumption peaks identified for all samples, which were characterized as the oxidation of the Mn^{2+} and Mn^{3+} species, respectively[3,12]. After Pd addition, the oxidation temperature of MnO slightly decreased from 270 °C to 240 °C. In principle, the higher oxidation temperature represented the weaker capability of oxygen activation, thus we can deduce that the activity of oxygen species for mullite was largely promoted by the addition of Pd. H_2-TPR and CO-TPR test had certified the existence of hydrogen spillover effect. Similarly, the oxygen spillover effect was affirmed as well in TPO test, interpreting the promoting effect of oxygen activity, as reported previously[3,13]. The Pd species could easily chemisorb gas-phase oxygen and serve as a donor for the catalyst, accelerating the oxygen mobility. Meanwhile, the reduced Mn species could accept the spilled oxygen species and accomplish an oxidation process. Synthetically considering the oxidation temperature during TPO process, the Pd/SMO-EG&M possessed the highest mobility of oxygen and would be favorable to serve satisfied catalytic efficiency.

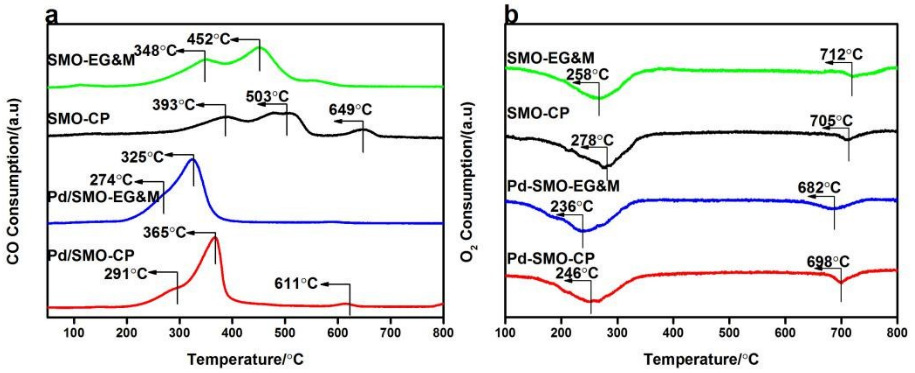

Fig. S6 (a) CO-TPR and (b) TPO profiles of the SMO and Pd/SMO.

S11. CO concentration during CO-TPD and CO_2-TPD

The concentration of desorbed CO was recorded with the m/z value of 28 in Fig. S7. Obviously, there's no desorption signal observed over all catalysts.

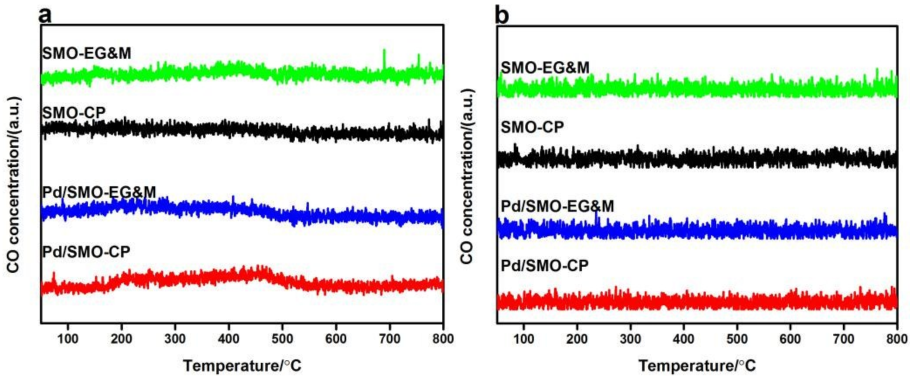

Fig. S7 MS signal of CO (28) in (a) CO-TPD and (b) CO_2-TPD profiles.

S12. Catalytic performance of individual oxide

Using $Pd(NO_3)_2$ (18.09 wt% in H_2O) as an additive, the reference $Sm(Mn,Pd)_2O_5$-CP (SMPO-CP) and $Sm(Mn,Pd)_2O_5$-EG&M (SMPO-EG&M) sample were prepared by adding $Pd(NO_3)_2$ to the precursors solutions to satisfy the same concentration. The content of Pd was set as 0.5% as well. As shown in Fig. S8, it is obvious that all Pd/SMO samples showed better performance than SMPO samples. Moreover, the SMPO samples showed just slightly enhanced, so it's supposed that the preparing method of Pd-mullite catalyst would largely effect the catalytic performance.

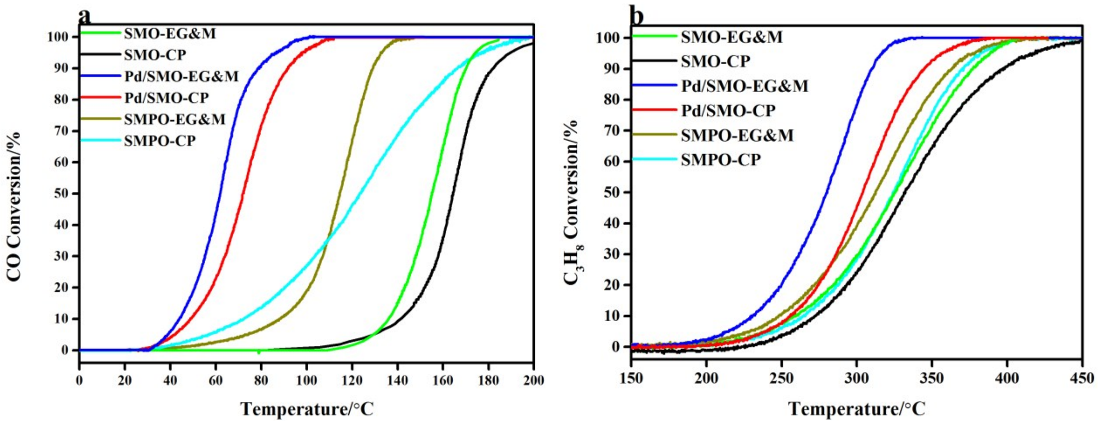

Fig. S8 Catalytic performance of: (a) CO oxidation; (b) C_3H_8 oxidation.

S13. Dynamic tests of CO and C_3H_8 oxidation

Kinetic study was conducted to calculate the reaction rate. According to our previous work[14], 25 mg meshed (80-100 mesh) catalysts were used to conduct CO and C_3H_8 oxidation kinetic study under the same gas components to eliminate the internal mass diffusion effect, and the flow rate was set as 400 ml/min to eliminate the external diffusion effect.The absolute reaction rates (r) were calculated using the Eq. S(4):

$$r\ \left(mol \cdot g^{-1}{}_{cat}\ s^{-1}\right) = V_{gas} \cdot C_{X,in} \cdot X / g_{cat} \qquad (4)$$

where V_{gas} is the reactant gas flow rate (mL/s) and $C_{X,in}$ is the feed concentration of X (mol/ml). g_{cat} is the weight of the catalyst employed (g). To determine the activation energy (E_a), the conversion was typically between 5% and 20%[15], and E_a was calculated from the Arrhenius plots of ln (r) - 1/T.

For Pd supported samples, the turnover frequency (TOF_{Pd}) was calculated using the Eq. S(5)

$$TOF_{Pd}(s^{-1}) = V_{gas} \cdot C_{X,in} \cdot X/N_{Pd} \cdot g_{Pd} \qquad (5)$$

where N_{Pd} and g_{Pd} represented the weight of Pd and molar amount of surface Pd sites per gram, respectively. The amount of active Pd site were determined by CO chemisorption[16]. At the same time, the TOF_{Mn} for SMO support was estimated as well according to molar amount of Mn. The relevant TOF values were summarized in Table S3.

Table S3 The TOF value for CO and C_3H_8 oxidation

Sample	CO oxidation at 100 ^{o}C		CO oxidation at 150 ^{o}C		C_3H_8 oxidation at 300 ^{o}C	
	TOF_{Pd} (10^{-1} s^{-1})	TOF_{Mn} (10^{-4} s^{-1})	TOF_{Pd} (10^{-2} s^{-1})	TOF_{Mn} (10^{-4} s^{-1})	TOF_{Pd} (10^{-2} s^{-1})	TOF_{Mn} (10^{-5} s^{-1})
SMO-EG&M	-	-	-	11.64	-	3.21
SMO-CP	-	-	-	6.17	-	1.69
Pd/SMO-EG&M	4.71	10.38	-	-	2.96	7.45
Pd/SMO-CP	5.33	6.44	-	-	3.68	4.45

S14. CO oxidation performance for different Pd catalysts

In a typical excess impregnation (EI) synthesis, the Pd/SMO-EG&M-EI was prepared with the final loading of 0.5 wt% according to previous report[16]. Besides, a hydrothermal (HT) synthesis of SMO nanorods was performed following the procedure reported by Liu[17]. However, the two catalysts don't display efficient catalytic performance as shown in Fig. S9. The Pd dispersion is low as shown in Table S4.

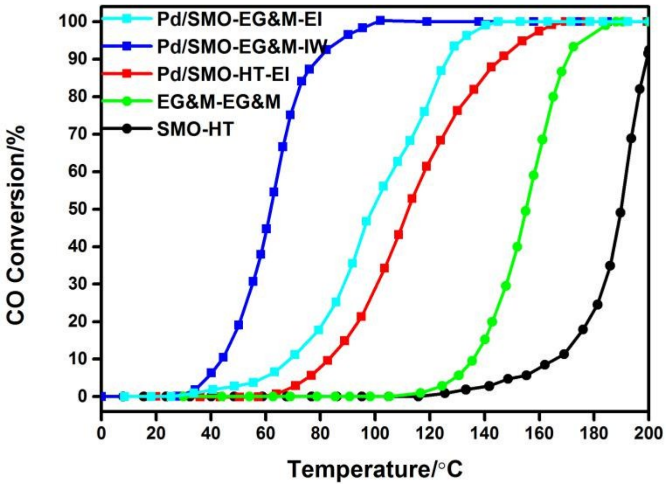

Fig. S9 CO oxidation activity of Pd/SMO catalysts prepared by different methods.

S15. Detailed characterizations of aged catalysts

The Pd catalysts aged at N_2 and H_2O were prepared to compare with that aged at O_2 and H_2O. The catalytic performance and physical characterizations were given below. It's obvious that the catalysts aged in N_2 suffer a dramatic loss of catalytic activity and the Pd oxide decompose while heating at 900 °C. The agglomeration of SMO support and Pd species are obvious, which lead to the loss of catalytic activity.

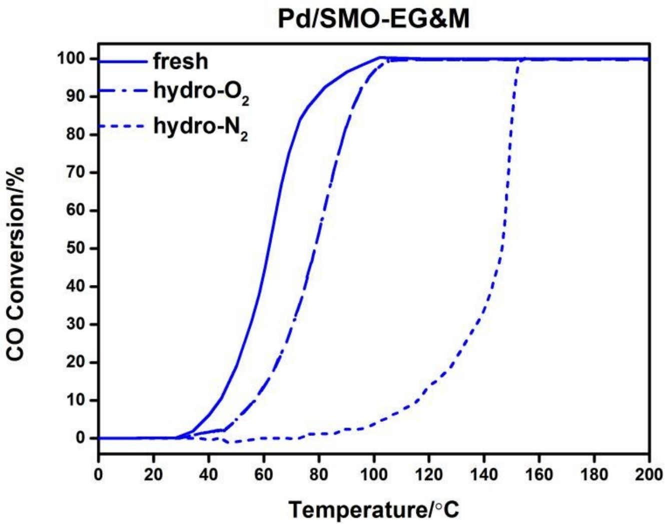

Fig. S10 CO oxidation performance for the fresh and aged Pd/SMO-EG&M.

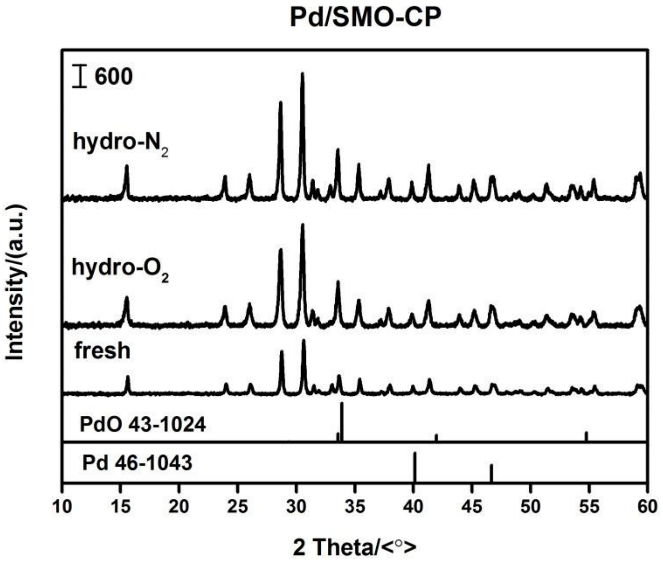

Fig. S11 XRD patterns of fresh and aged Pd/SMO catalysts.

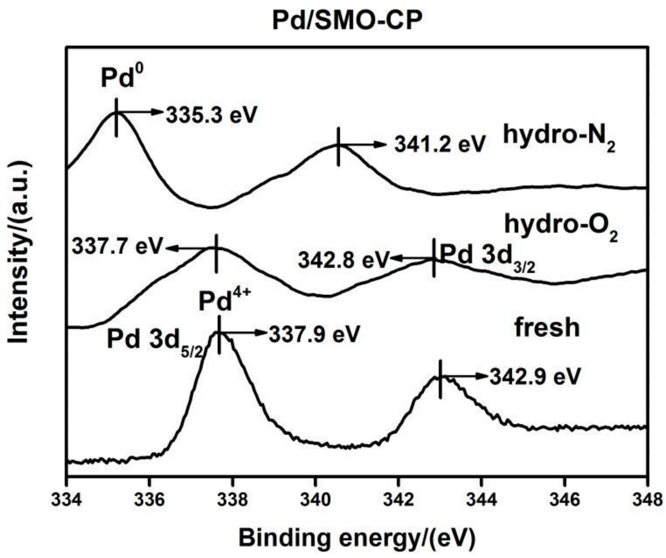

Fig. S12 Pd 3d XPS spectra of fresh and aged Pd/SMO-CP catalysts.

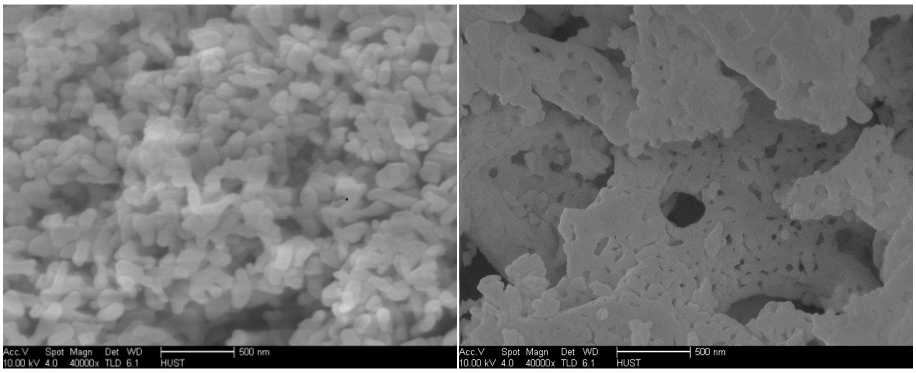

Fig. S13 SEM image of (a) Pd/SMO-CP and (b) Pd/SMO-EG&M.

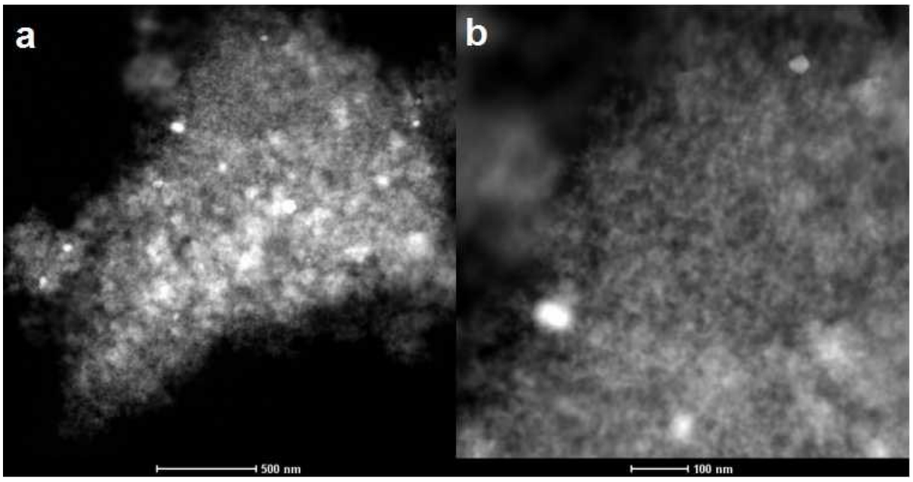

Fig. S14 STEM images of aged Pd/SMO-CP catalyst.

Table S4 The Pd dispersion of other Pd catalysts and the aged samples

Catalysts	Surface coverage ($\mu mol/g_{cat}$)	Pd dispersion (%)	Pd size (nm)
Pd/SMO-HT	3.9±0.5	8.4±1.0	11.6-14.8
Pd/SMO-EG&M-IW	4.9±0.4	10.4±0.9	9.6-11.4
Pd/SMO-EG&M-aged	9.1±0.6	19.3±1.3	5.3-6.0
Pd/SMO-CP-aged	4.3±0.4	9.1±0.9	10.8-13.3

Reference

[1] H. X. Mai, L. D. Sun, Y. W. Zhang, R. Si, W. Feng, H. P. Zhang, H. C. Zhang and C. H. Yan, J. Phy. Chem. B, 2005, 109, 24380-24385.

[2] J. H. Chen, M. Q. Shen, X. Q. Wang, J. Wang, Y. G. Su and Z. Zhao, Catal. Commu., 2013, 37, 105-108.

[3] J. Y. Luo, M. Meng, J. S. Yang, X. G. Li, Y. Q. Zha, X. T. Wang and T. Y. Zhang, Appl. Catal., B, 2009, 87, 92.

[4] S. H. Liang, T. G. Xu, F. Teng, R. L. Zong and Y. F. Zhu, Appl. Catal., B, 2010, 96, 267-275.

[5] Tana, M. L. Zhang, J. Li, H. J. Li, Y. Li and W. J. Shen, Catal. Today, 2009, 148, 179-183.

[6] J. Wang, Y. G . Su, X. Q . Wang, J. H. Chen, Z. Zhao and M. Q. Shen, Catal. Commun., 2012, 25, 106-109.

[7] Y. Hasegawa, K. Fukumoto, T. Ishima, H. Yamamoto, M. Sano and T. Miyake, Appl. Catal., B, 2009, 89, 420-424.

[8] J. Y. Luo, D. Kisinger, A. Abedi and William S. Epling, Appl. Catal., A, 2010,

383, 182.

[9] H. Sharma and A. Mhadeshwar, Appl. Catal., B, 2012, 127, 190.

[10] Z. Q. Zou, M. Ming and Y. Q. Zha, J. Phys. Chem. C, 2010, 114, 468-477.

[11] J. Y. Luo, M. Meng, Y. Q. Zha, and L. H. Guo, J. Phys. Chem. C, 2008, 112, 8694-8701.

[12] Q. Liang, X. D. Wu, D. Weng and H. B. Xu, Catal. Today, 2008, 109, 113-118.

[13] K.i Ramesh, L. W. Chen, F. X. Chen, Y. Liu, Z. Wang and Y. F. Han, Catal. Today, 2008, 131, 477-482.

[14] Z. J. Feng, J. Q. Wang, X. Liu, Y. W. Wen, R. Chen, H. F. Yin, M. Q. Shen and B. Shan, Catal. Sci. Techno., 2016, 6, 5580-5589.

[15] Z. Hu, X. F. Liu, D. M. Meng, Y. Guo, Y. L. Guo and G. Z. Lu, ACS Catal., 2016, 6, 2265−2279.

[16] G, Spezzati, Y. Q. Su, J P. Hofmann, A D. Benavidez, A T. DeLaRiva, J. McCabe, A K. Datye and E J. M. Hensen, ACS Catal., 2017, 7, 6887-6891.

[17] J. Y. Liu, M. Yu, X. W. Wang, J. Wu, C. H. Wang, L. J. Zheng, D. C. Yang, H. Liu, Y. Yao, F. Lu and W. C. Wang, J. Mater. Chem. A, 2017, 5, 20922.
